# Antigen-Presenting Cancer-Associated Fibroblasts Modulate Tumor Microimmunity

**DOI:** 10.31662/jmaj.2026-0126

**Published:** 2026-08-21

**Authors:** Yasuhiro Fukui, Hiroaki Kasashima, Nobuhiro Naito, Zizhou Wang, Iguru Omori, Yuki Seki, Masatsune Shibutani, Yu Muta, Yuki Nakanishi, Masakazu Yashiro, Naoko Ohtani, Kiyoshi Maeda

**Affiliations:** 1 Department of Gastroenterological Surgery, Osaka Metropolitan University, Graduate School of Medicine, Osaka, Japan; 2 Department of Gastroenterology and Hepatology, Kyoto University, Graduate School of Medicine, Kyoto, Japan; 3 Department of Molecular Oncology and Therapeutics, Osaka Metropolitan University, Graduate School of Medicine, Osaka, Japan; 4 Department of Pathophysiology, Osaka Metropolitan University, Graduate School of Medicine, Osaka, Japan

**Keywords:** antigen-presenting cancer-associated fibroblast, tumor microenvironment, mesothelial cell, fibrocyte, antigen presentation

## Abstract

Cancer-associated fibroblasts (CAFs) are key regulators of tumor progression, immune modulation, and therapeutic resistance within the tumor microenvironment. Advances in single-cell and spatial profiling have revealed substantial heterogeneity among CAF populations, challenging the traditional view of CAFs as a uniformly tumor-promoting stromal population. Among the identified fibroblast states, antigen-presenting CAFs (apCAFs) represent a distinct subset characterized by major histocompatibility complex class II expression and the ability to directly interact with cluster of differentiation (CD) 4^+^ T cells.

First described in pancreatic ductal adenocarcinoma, apCAFs have since been observed in multiple tumor types. Unlike professional antigen-presenting cells, apCAFs lack classical costimulatory molecules, suggesting that their antigen presentation leads to non-canonical immune outcomes, including immune modulation, immune tolerance, or regulatory T cell induction. Recent evidence indicates that apCAFs arise from multiple cellular sources, most prominently mesothelial cells undergoing mesothelial-to-mesenchymal transition in response to tumor-derived signals such as interleukin-1 and transforming growth factor-β. Additional studies have identified fibrocyte-like apCAFs derived from bone marrow–derived cells, underscoring the heterogeneous and context-dependent nature of this fibroblast state.

Consistent with their antigen-presenting capacity, apCAFs localize to immune-enriched niches, including tertiary lymphoid structures, where they influence local immune architecture and T cell functional states. Notably, apCAFs have been linked to both tumor-promoting and tumor-restraining activities, highlighting their dual roles in cancer immunity. Importantly, these immunological functions have direct clinical implications: apCAF-enriched stromal programs are associated with distinct immune landscapes and may influence responses to immune checkpoint blockade.

This review summarizes current knowledge of the biology of apCAFs, with emphasis on their defining molecular features, cellular origins, immunological functions, and clinical relevance. Therefore, defining the context-dependent roles of apCAFs will be essential for designing next-generation stromal-targeted and immunotherapy-combination strategies that selectively enhance antitumor immunity while limiting tumor-promoting fibroblast functions.

## Introduction

Cancer-associated fibroblasts (CAFs) are a key stromal component of the tumor microenvironment (TME) that regulate tumor progression, modulate immune responses, and influence therapeutic outcomes ^(1), (2), (3)^. The critical role of the TME has been extensively investigated. These studies reveal intricate interactions between cancer cells and the surrounding stromal components and demonstrate how these interactions influence patient prognosis across cancer types. In addition to malignant epithelial cells, tumors contain diverse stromal and immune elements; among these, CAFs are one of the most abundant populations and play central regulatory roles. They exert broad regulatory effects through extracellular matrix (ECM) remodeling, cytokine secretion, and immune modulation ^(2), (4), (5)^. Clinically, a fibroblast-rich or desmoplastic tumor stroma is frequently associated with aggressive disease and poor prognosis across multiple cancer types ^(6), (7), (8), (9), (10), (11), (12)^.

CAFs are no longer considered a homogeneous population with uniformly tumor-promoting functions. Recent advances in single-cell transcriptomics and spatial profiling have revealed pronounced CAF heterogeneity and enabled classification into distinct subsets, including myofibroblastic CAFs (myCAFs) and inflammatory CAFs (iCAFs) ^(13), (14), (15), (16)^. These subsets differ in molecular signatures, spatial localization, and functional roles within the TME. The experimental depletion of CAFs has yielded tumor-accelerating effects in some models, underscoring the existence of tumor-restraining fibroblast states and highlighting the complexity of stromal-targeting strategies ^(15)^.

Within this framework of CAF heterogeneity, antigen-presenting CAFs (apCAFs) have emerged as a distinct and conceptually transformative subset. apCAFs are characterized by the expression of major histocompatibility complex class II (MHC-II)–related molecules, including cluster of differentiation (CD)74 and human leukocyte antigen (HLA) class II genes, enabling direct interaction with CD4^+^ T cells ^(14)^. First identified in pancreatic ductal adenocarcinoma (PDAC), apCAFs have since been reported across multiple tumor types, challenging the paradigm that antigen presentation in tumors is restricted to professional antigen-presenting cells.

Spatial transcriptomic and imaging analyses demonstrate that apCAFs frequently localize to immune-enriched niches, including tertiary lymphoid structures (TLSs). In these regions, they influence local T cell activation, differentiation, and immune organization ^(17)^. Because TLSs are associated with improved responses to immune checkpoint blockade, the presence of apCAFs in these niches suggests a potential role in shaping immunotherapy outcomes. However, the functional consequences of antigen presentation by fibroblasts appear context dependent, with evidence supporting both immunomodulatory and potentially immunosuppressive roles ^(17), (18), (19)^. Moreover, the cellular origins of apCAFs—whether derived from resident fibroblasts, mesothelial cells, or bone marrow–derived precursors or induced through phenotypic reprogramming by tumor-derived signals—remain incompletely understood.

This review summarizes current insights into apCAFs, focusing on their emergence within CAF heterogeneity, their defining molecular features and cellular origins, and their roles as modulators of tumor immunity (Figure 1). We also discuss the clinical and therapeutic implications of apCAFs, particularly in the context of cancer immunotherapy, and outline key challenges and future directions for translating apCAF biology into clinical benefit.

**Figure 1.**
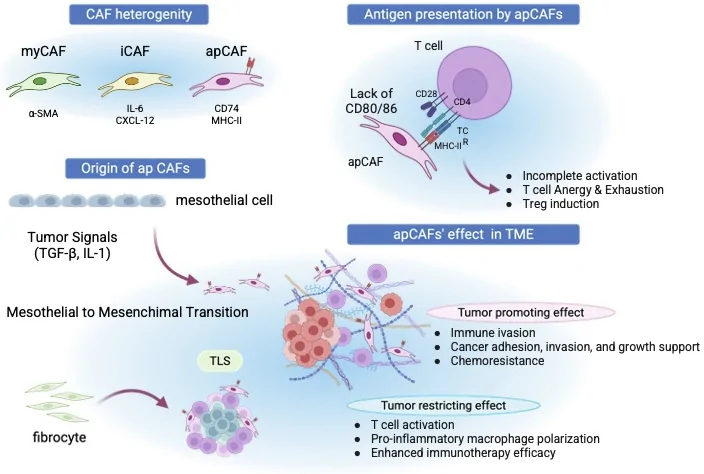
Schematic representation of apCAFs in the tumor microenvironment. Tumor-derived signals induce mesothelial cells to undergo mesothelial-to-mesenchymal transition, generating MHC class II–expressing apCAFs that mediate immunosuppressive effects through incomplete antigen presentation. Fibrocyte-derived apCAFs localize within tertiary lymphoid structures. In contrast, apCAFs can also exert immune-supportive functions and promote antitumor immunity, highlighting the functional heterogeneity of the apCAF population. apCAF: antigen-presenting cancer-associated fibroblast; MHC: major histocompatibility complex.

## CAF Heterogeneity and the Emergence of apCAFs

CAFs have historically been viewed as a relatively uniform stromal population that promotes tumor growth through ECM remodeling and paracrine signaling ^(20), (21), (22)^. However, this view has been fundamentally revised over the past decade with the advent of high-resolution transcriptomic and spatial profiling technologies. Single-cell RNA sequencing (scRNA-seq) studies across multiple cancer types have consistently demonstrated that CAFs comprise transcriptionally and functionally distinct subpopulations, reflecting both lineage diversity and dynamic phenotypic plasticity within the TME ^(2), (14), (15), (23)^.

One of the earliest and most influential frameworks for CAF heterogeneity emerged from studies in PDAC, which identified two major CAF subsets: myCAFs and iCAFs ^(13)^. myCAFs are characterized by high expression of contractile and ECM-related genes—such as *ACTA2* (α-smooth muscle actin [αSMA]) and *COL1A1*—and are typically localized in close proximity to tumor cells. In contrast, iCAFs exhibit low αSMA expression but secrete high levels of inflammatory cytokines and chemokines, including interleukin-6 (IL-6) and C-X-C motif chemokine ligand 12, and tend to localize more distally within the stromal compartment ^(13), (24)^. Subsequent pan-cancer analyses further expanded the CAF landscape, revealing additional fibroblast states associated with interferon signaling, antigen processing, metabolic regulation, and immune modulation ^(2), (14), (24)^. Importantly, experimental studies demonstrated that CAF phenotypes are highly plastic and can dynamically interconvert in response to tumor-derived factors, cytokine gradients, and mechanical signals within the TME. These findings have shifted the prevailing paradigm from a lineage-based classification to a state-based model, in which CAF heterogeneity reflects context-dependent transcriptional programs rather than fixed cellular identities.

Within this broader framework of CAF heterogeneity, a subset of fibroblasts expressing genes related to antigen processing and presentation has been reproducibly identified across multiple scRNA-seq datasets ^(14)^. The recognition of this apCAF state represents a logical extension of the evolving understanding of CAF diversity, particularly at the interface between stromal biology and tumor immunity. Rather than representing an isolated or anomalous population, apCAFs can be viewed as part of a continuum of fibroblast states that emerge under specific immunological and microenvironmental conditions.

Thus, the concept of apCAFs is best understood within the context of CAF heterogeneity and plasticity. This perspective provides a conceptual foundation for examining how apCAFs are defined at the molecular level, how they arise within tumors, and how they influence immune responses, as discussed in the following sections.

## Defining Features and Origins of apCAFs

apCAFs represent a distinct fibroblast state defined by the expression of genes involved in antigen processing and presentation, most notably MHC-II–related molecules. Transcriptionally, apCAFs are characterized by high expression of CD74 and HLA class II genes (e.g., *HLA-DRA* and *HLA-DRB1*), while lacking classical costimulatory molecules such as CD80 and CD86 that are typically required for full T cell activation by professional antigen-presenting cells ^(14), (25), (26)^. Table 1 lists representative marker genes reported for apCAFs. This unique molecular signature distinguishes apCAFs from conventional myCAF and iCAF subsets and suggests a specialized role at the interface of stromal biology and tumor immunity.

**Table 1.** Representative Marker Genes of Antigen-Presenting Cancer-Associated Fibroblasts in Human and Mouse.

Human		
No.	Gene	Description
1	CD74 (cluster of differentiation 74 [MHC class II invariant chain])	MHC class II chaperone required for antigen presentation
2	HLA-DRA (human leukocyte antigen DR alpha)	MHC class II alpha chain involved in CD4^+^ T cell antigen presentation
3	HLA-DRB1 (human leukocyte antigen DR beta)	MHC class II beta chain of the antigen-presenting complex
4	HLA-DQA1 (human leukocyte antigen DQ alpha 1)	MHC class II DQ alpha chain involved in adaptive immunity
5	SAA3 (serum amyloid A3)	Inflammation-induced acute-phase protein
6	SLPI (secretory leukocyte peptidase inhibitor)	Secreted protease inhibitor that regulates inflammation and tumor–stroma interactions
7	MSLN (mesothelin)	Mesothelial cell surface protein associated with tumor–stroma adhesion
8	UPK3B (uroplakin 3B)	Mesothelial-associated membrane protein
9	CD24	Cell surface glycoprotein involved in immune evasion and adhesion
10	CA9 (carbonic anhydrase IX)	Hypoxia-inducible enzyme involved in pH regulation
11	EGLN3 (Egl-9 family hypoxia inducible factor 3; also known as prolyl hydroxylase domain protein 3 [PHD3])	Oxygen-sensing enzyme controlling HIF signaling
12	C1QC (complement C1q subcomponent subunit C)	Complement component commonly expressed by myeloid-lineage cells
13	APOC1 (apolipoprotein C1)	Myeloid-associated lipid metabolism protein with immunoregulatory functions
14	RARRES1 (retinoic acid receptor responder 1)	Fibroblast activation–associated retinoic acid–responsive gene
15	PTPRC (protein tyrosine phosphatase receptor type C [CD45])	Pan-leukocyte marker indicating immune cell origin
16	CD37	Immune cell tetraspanin involved in signal transduction
17	CD52	Lymphocyte surface antigen involved in immune regulation
18	SPP1 (secreted phosphoprotein 1 [osteopontin])	Secreted ECM protein that promotes immune modulation and tumor progression
**Mouse**		
**No.**	**Gene**	**Description**
19	CD74	MHC class II chaperone required for antigen presentation
20	H2-Aa (histocompatibility 2, class II antigen A, alpha chain [mouse MHC class II α chain])	MHC class II alpha chain for CD4^+^ T cell antigen presentation
21	H2-Ab1 (histocompatibility 2, class II antigen A, beta 1 chain [mouse MHC class II β chain])	MHC class II beta chain forming the antigen-presenting complex
22	Slpi (secretory leukocyte peptidase inhibitor)	Secreted protease inhibitor that regulates inflammation and stromal–epithelial interactions
23	Saa3 (serum amyloid A3)	Inflammation-induced acute-phase protein
24	Nkain4 (Na^+^/K^+^ transporting ATPase interacting 4)	Mesothelial-associated membrane protein involved in cell adhesion
25	Irf5 (interferon regulatory factor 5)	Transcription factor that regulates inflammatory and immune gene expression
26	Msln (mesothelin)	Mesothelial cell surface protein associated with tumor–stroma interactions
27	Upk3b (uroplakin 3B)	Mesothelial-associated membrane protein

ECM: extracellular matrix; MHC: major histocompatibility complex.

Functionally, the antigen-presenting capacity of apCAFs differs fundamentally from that of dendritic cells or macrophages. The absence of costimulatory signals implies that their interactions with CD4^+^ T cells may lead to alternative immunological outcomes, including partial activation, tolerance, or modulation of T cell differentiation rather than robust effector responses. This atypical antigen-presenting phenotype highlights the need to consider apCAFs as non-canonical regulators of adaptive immunity within the TME.

The initial identification of apCAFs in PDAC by scRNA-seq analyses revealed a fibroblast population expressing MHC-II genes and exhibiting transcriptional similarities to mesothelial cells. These observations raised the possibility that apCAFs may arise from a mesothelial lineage rather than from conventional tissue-resident fibroblasts. Supporting this hypothesis, mesothelial markers such as Wilms tumor 1 and mesothelin (MSLN) were found to be co-expressed with antigen-presentation-related genes in subsets of CAFs. Lineage-tracing experiments in mouse models further demonstrated that mesothelial cells can undergo phenotypic transition and contribute to the CAF pool during tumor progression ^(27)^. In vitro experiments further showed that IL-1 and transforming growth factor-β (TGF-β) signaling pathways induce the transition of mesothelial cells into apCAF-like cells. Consistent with these findings, mesothelial cell-derived CAFs have been observed in peritoneal dissemination lesions, and mesothelial cells are known to undergo mesothelial-to-mesenchymal transition (MMT) in response to TGF-β signaling ^(28), (29)^. Recent studies indicate that mesothelial cells are not merely passive barriers lining the serosal cavities; they actively shape the TME through dynamic interactions with immune cells. Mesothelial cells secrete cytokines, chemokines, and extracellular vesicles that recruit immunosuppressive myeloid cells and modulate T cell responses, thereby promoting tumor progression and immune evasion ^(30)^. Mesothelial cell–derived CAFs subsequently assume typical CAF functions and contribute to the establishment of a permissive niche for cancer-cell adhesion, invasion, and growth.

In addition to mesothelial-associated apCAFs (M-apCAFs), an alternative origin of apCAFs has been proposed based on the identification of fibrocyte-associated apCAF (F-apCAF) populations ^(18)^. Fibrocytes are derived from bone marrow–derived hematopoietic precursors and are characterized by the co-expression of hematopoietic markers, such as CD45, and mesenchymal or fibroblastic markers, including ECM proteins ^(31), (32)^. Functionally, fibrocytes are known to migrate to sites of tissue injury, where they contribute to inflammatory responses and fibrotic remodeling ^(33)^, and have therefore been implicated in chronic inflammation, fibrosis, and tumor-associated stromal reactions. Consistent with this definition, Chen et al ^(18)^ reported that F-apCAFs express multiple hematopoietic genes, including *PTPRC* (encoding CD45), *CD37*, and *CD52*, in addition to antigen presentation–related genes. The coexistence of hematopoietic and fibroblastic transcriptional programs distinguishes the F-apCAF subset from both M-apCAFs and conventional resident fibroblast–derived CAFs. These findings suggest that a fraction of apCAFs may originate from circulating or bone marrow–derived fibrocyte-like cells that acquire antigen-presenting capacity within the TME.

Collectively, current evidence indicates that apCAFs are defined by a combination of molecular features—MHC-II expression in the absence of costimulation—and context-dependent cellular origins. Rather than representing a single lineage-derived population, apCAFs likely comprise a spectrum of fibroblast states that converge on an antigen-presenting phenotype in response to specific immunological and microenvironmental cues. This conceptual framework provides a basis for understanding the diverse immunological roles of apCAFs and sets the stage for examining in the following section how these cells modulate tumor immunity.

## apCAFs as Modulators of Tumor Immunity

Functional studies indicate that apCAFs exert context-dependent effects on tumor immunity, with evidence supporting both immunosuppressive and immune-supportive roles. apCAFs occupy a unique position within the TME through direct interaction with adaptive immune cells. Unlike conventional CAF subsets, apCAFs express MHC-II molecules and can present antigenic peptides to CD4^+^ T cells. Seminal single-cell transcriptomic analyses and functional assays demonstrated that apCAFs can process and present antigens in an MHC-II–dependent manner, establishing fibroblasts as non-canonical antigen-presenting cells within tumors ^(14)^. This finding expanded the concept of stromal-immune crosstalk and provided a mechanistic basis for the immunomodulatory roles of CAFs.

### Tumor-promoting immunological functions of apCAFs

Accumulating evidence indicates that apCAFs can promote tumor progression by shaping immunosuppressive T cell responses. A key mechanism involves the induction of regulatory T cells (Tregs), as apCAFs present antigens to naïve CD4^+^ T cells in the absence of classical costimulatory signals, thereby favoring Treg differentiation over effector T cell activation ^(27)^. This mode of antigen presentation contributes to local immune tolerance and facilitates tumor immune evasion. Studies on M-apCAFs demonstrated that blocking the mesothelial cell–to-apCAF transition has profound immunological consequences. Treatment with anti-MSLN antibodies inhibited the differentiation of mesothelial cells into apCAFs, resulting in increased intratumoral CD8^+^ T cell infiltration and induced significant tumor regression in preclinical models, indicating that apCAFs contribute to an immunosuppressive TME that restrains cytotoxic T cell–mediated antitumor immunity.

In gastric cancer, tumor-derived TGF-β and IL-1 induce MMT. Mesothelial cells that acquire mesenchymal characteristics recruit immunosuppressive myeloid cells via chemokine secretion. They also facilitate peritoneal attachment of gastric cancer cells by producing tenascin C as a structural scaffold, thereby promoting peritoneal dissemination ^(34)^.

Recent studies further show that apCAFs are enriched within secreted phosphoprotein 1 (SPP1)–positive stromal niches in PDAC. In this context, apCAF-derived SPP1 promotes tumor progression and confers resistance to chemotherapy. Although myeloid cells and T cells are present in M-apCAF niches, the infiltrating T cells predominantly display exhausted and immunosuppressive phenotypes, supporting the notion that M-apCAFs contribute to localized immune suppression rather than effective immune activation. Notably, F-apCAFs, which localize to lymphocyte-enriched niches, are also associated with poor prognosis, as observed for M-apCAFs ^(18)^.

### Tumor-restricting and immune-supportive functions of apCAFs

In contrast to their immunosuppressive roles, emerging data indicate that apCAFs can support antitumor immunity in specific spatial and immunological contexts. Single-cell analyses in PDAC identified highly proliferative mesenchymal subsets, including MHC class II– and CD74-expressing populations, that were enriched within CD105-negative (CD105^neg^) CAFs ^(35)^. Genes associated with MHC class II antigen presentation were predominantly expressed in CD105^neg^ CAFs, indicating substantial transcriptional overlap with apCAFs. Notably, CD105^neg^ CAFs were almost uniquely characterized by in vivo expression of MHC-II antigen presentation machinery and could be induced to express these molecules in vitro. However, genetic disruption of MHC-II, CD74, or CD80 in CD105^neg^ pancreatic fibroblasts did not abolish their tumor-restrictive activity in vivo. These findings indicate that, although CD105^neg^ CAFs overlap transcriptionally with apCAFs, their antitumor function is independent of classical fibroblast-mediated antigen presentation.

Large-scale spatial multi-omics analyses identified a CAF subtype closely overlapping with apCAFs, termed s4-CAFs, that localizes to lymphoid aggregates and TLSs ^(17)^. These cells interact most strongly with B cells, followed by T cells and plasma cells, suggesting a role in organizing local adaptive immune responses. Mechanistically, s4-CAFs express chemokines such as C-C motif chemokine ligand 19 (CCL19) and CCL21, which recruit C-C motif chemokine receptor 7–expressing T cells and B cells and may promote the formation and maintenance of lymphoid aggregates and TLSs.

Additional evidence for the tumor-restrictive activity of apCAFs comes from studies in lung cancer, where MHC-II–expressing fibroblasts were shown to rescue intratumoral CD4^+^ T cells from apoptosis via signaling through the C1q receptor C1qbp ^(36)^. This survival support for CD4^+^ T cells was essential to sustain effective antitumor immunity, highlighting a context-dependent protective function of apCAFs in the TME.

Collectively, these findings indicate that both mesothelial-derived apCAFs and TLS-enriched apCAFs represent heterogeneous subpopulations with context-dependent and potentially opposing immunological functions. This functional heterogeneity has direct implications for therapeutic targeting and for predicting response to immunotherapy in apCAF-enriched tumors.

## Clinical and Therapeutic Implications of apCAFs

The identification of apCAFs has important clinical implications that extend beyond their biological roles within the TME. Previous studies highlight their therapeutic relevance, particularly in the context of tumor immunity and resistance to immunotherapy.

Several studies suggest that apCAFs may contribute to resistance to immune checkpoint blockade ^(18), (27)^. Incomplete antigen presentation by apCAFs is proposed to induce T cell dysfunction rather than effective activation. This tolerogenic interaction may persist in tumors treated with immune checkpoint inhibitors, thereby limiting the efficacy of therapies targeting the PD-1/PD-L1 pathways. These observations indicate that immune checkpoint blockade alone may be insufficient in apCAF-enriched tumors, as stromal antigen presentation can maintain local immune suppression independently of tumor cell–intrinsic immune evasion mechanisms.

Conversely, accumulating evidence indicates that apCAF-enriched tumors display immune-inflamed microenvironments, enhanced antigen presentation, and improved responsiveness to immune checkpoint inhibitors ^(19)^. In this context, apCAFs may serve as biomarkers of functional immune niches rather than as uniformly immunosuppressive stromal cells.

These findings underscore a key limitation of strategies that broadly target or deplete CAFs. Given their marked heterogeneity, non-selective stromal targeting may fail to suppress tumor-promoting fibroblast programs and instead remove fibroblast subsets that support antitumor immunity ^(37)^. Moreover, apCAFs themselves can exert both tumor-promoting and tumor-restraining effects, indicating that indiscriminate targeting of MHC class II–expressing apCAFs risks loss of beneficial immune-supportive functions.

The clinical relevance of apCAFs therefore lies in their ability to delineate stromal states that are permissive or restrictive to antitumor immunity, informing patient stratification and treatment selection. Beyond their biomarker potential, specific apCAF-associated programs may be therapeutically targetable. In particular, subsets of apCAFs form tumor-promoting immune niches through the expression of SPP1 and cooperation with myeloid cells to support tumor progression, metastasis, and treatment resistance. Experimental disruption of SPP1-driven stromal programs attenuates tumor growth and partially restores antitumor immune activity, suggesting that selective targeting of SPP1-expressing apCAF niches could dismantle protumorigenic stromal circuits without global fibroblast depletion ^(18)^.

Collectively, these findings support a shift from fibroblast ablation to precision stromal modulation, in which apCAFs function both as indicators of immune context and as targets for selective therapeutic intervention. Clinical strategies that integrate spatial profiling with functional characterization of apCAFs may enable more effective combination therapies that suppress tumor-promoting stromal programs while preserving or enhancing immune-supportive fibroblast functions.

## Conclusions

apCAFs have emerged as a distinct and functionally important CAF state linking stromal biology to tumor immunity. First identified through high-resolution single-cell analyses, apCAFs challenge the traditional view of CAFs as uniformly immunosuppressive cells by demonstrating that fibroblasts can directly participate in antigen presentation and immune regulation within the TME.

Emerging evidence indicates that apCAFs are not a uniform population. Instead, they comprise a spectrum of states shaped by cellular origin, spatial context, and microenvironmental cues. Mesothelial-derived apCAFs, fibrocyte-like apCAFs, and apCAF-like subsets within TLSs illustrate how antigen-presenting capacity can arise through distinct developmental or inducible pathways. The immunological consequences of apCAF activity are highly context dependent and induce tumor-promoting mechanisms, such as Treg induction and the formation of protumorigenic niches, as well as tumor-restraining functions that support adaptive immune responses.

From a clinical perspective, apCAF heterogeneity has important implications. apCAFs do not serve as uniform therapeutic targets; instead, they reflect immune contexture and stromal states that influence treatment responsiveness. Recent studies suggest that apCAF-enriched tumors are more likely to exhibit immune-inflamed phenotypes and respond to immunotherapy, highlighting their potential as biomarkers. Targeting tumor-promoting apCAF programs, such as SPP1-driven stromal niches, directly translates mechanistic insights into therapy by suppressing tumor progression and overcoming treatment resistance while avoiding indiscriminate fibroblast depletion.

In summary, apCAFs represent a conceptual shift in our understanding of fibroblast biology in cancer, emphasizing functional plasticity over static lineage definitions. Future studies integrating spatial multi-omics, functional perturbation, and clinical trial data will be essential to define the regulation of apCAF states. These insights will inform therapeutic strategies for precision stromal modulation. Such approaches may enable strategies that reshape, rather than eradicate, the tumor stroma to promote durable antitumor immunity.

## Article Information

### Acknowledgments

We thank Akiko Tsuda (Osaka Metropolitan University, Graduate School of Medicine) for technical assistance. We would like to thank Editage (www.editage.jp) for English language editing.

### Author Contributions

Conceptualization: Yasuhiro Fukui and Hiroaki Kasashima. Writing – original draft preparation: Yasuhiro Fukui. Writing – review and editing: Hiroaki Kasashima, Nobuhiro Naito, Zizhou Wang, and Iguru Omori. Funding acquisition: Hiroaki Kasashima. Resources: Hiroaki Kasashima and Kiyoshi Maeda. Supervision: Yuki Seki, Masatsune Shibutani, Yu Muta, Yuki Nakanishi, Masakazu Yashiro, Naoko Ohtani, and Kiyoshi Maeda. All authors have read and agreed to the published version of the manuscript.

### Conflicts of Interest

None

### Institutional Review Board Approval Code and Number

Not applicable.

### Article Information

This article is based on the study that received the Medical Research Encouragement Prize of the Japan Medical Association in 2025.
